# Exercise in Postural Orthostatic Tachycardia Syndrome: Focus on Individualized Exercise Approach

**DOI:** 10.3390/jcm13226747

**Published:** 2024-11-09

**Authors:** Kristine Zeznick Trimble, Jennifer N. Switzer, Svetlana Blitshteyn

**Affiliations:** 1 Dysautonomia Clinic, Williamsville, NY 14221, USA; 2 Department of Neurology, University at Buffalo Jacobs School of Medicine and Biomedical Sciences, Buffalo, NY 14203, USA

**Keywords:** postural orthostatic tachycardia syndrome, dysautonomia, orthostatic intolerance, exercise, physical therapy, rehabilitation, remote, long COVID, hypermobile Ehler–Danlos syndrome, mast cell activation syndrome, hypermobility spectrum disorders

## Abstract

Exercise is a vital component of health and is commonly utilized as a non-pharmacologic therapy for many disorders, including postural orthostatic tachycardia syndrome (POTS). However, exercise intolerance is a key feature of POTS and other autonomic disorders and, therefore, presents a major barrier for many patients. Despite exercise being uniformly recommended as a therapeutic intervention, a majority of patients with POTS, especially those with severe orthostatic intolerance and fatigue, are unable to complete or sustain rigorous exercise programs or successfully integrate them into their daily routine. In this narrative review, we discuss the current literature on exercise and POTS and our clinical experience with a home-based exercise approach developed at the Dysautonomia Clinic. We conclude that individualized exercise programs that are delivered remotely by a certified physical therapist may be convenient, easily accessible, and safe for patients with POTS, especially those with severe symptoms who may be home- or bedbound. Future randomized controlled studies are needed to quantify and characterize the benefits of home-based exercise programs delivered remotely compared to standard therapy.

## 1. Introduction

The autonomic nervous system (ANS) consists of sympathetic, parasympathetic, and enteric divisions and is responsible for numerous physiologic functions, including cardiovascular control of the heart rate and blood pressure, gastric motility and secretion, bladder function, respiration, temperature control, and distribution of blood flow to organs and tissue. The ANS mediates the “flight or fight” response to both external and internal stimuli in order to maintain homeostasis. Consequently, since exercise involves a complex interplay between cardiovascular, pulmonary, metabolic, and neurologic systems to increase cardiac output and blood flow, disorders of the autonomic nervous system would involve altered physiologic responses to exercise, resulting in exercise intolerance.

One of the most common autonomic disorders, postural orthostatic tachycardia syndrome (POTS), is characterized by postural tachycardia, orthostatic intolerance, dizziness, fatigue, and headache, along with several other symptoms. An estimated 3 million people in the United States had POTS before the COVID-19 pandemic, but the prevalence is now significantly higher and has likely at least doubled, due, in part, to POTS that can follow SARS-CoV-2 infection as part of Long COVID [1]. Other triggers of POTS include surgery, pregnancy, menarche, vaccination, or concussion [2].

The diagnostic criteria for POTS are as follows: (1) an increase in heart rate of at least 30 beats per minute in adults and at least 40 beats per minute in teens up to 19 years of age within 10 min of standing or during a tilt table test; (2) absence of orthostatic hypotension; and (3) symptoms of orthostatic intolerance present for at least six months [3]. Other conditions that can mimic POTS, such as infection, dehydration, severe anemia, prolonged bed rest, hyperthyroidism, anorexia nervosa, and pheochromocytoma, need to be excluded [3].

Several possible pathophysiologic mechanisms were proposed in the early 1990s when POTS was first described. Cardiac deconditioning was among these, when it was observed that patients with POTS were performing at a lower level of cardiac performance due to reduced stroke volume and VO2 max than their healthy counterparts [4]. It was then suggested that, if cardiac deconditioning is the cause of POTS, then exercise may effectively treat or even cure it. Subsequent studies using invasive cardiopulmonary testing in POTS and, more recently, in Long COVID demonstrated that cardiac deconditioning was not the primary underlying mechanism and that other physiologic factors, such as cerebral hypoperfusion, decreased peripheral oxygen extraction, reduced venous return with low ventricular filling pressures, and others, are likely responsible for exercise intolerance [5,6,7]. Although somatic hypervigilance and anxiety were suggested as being prevalent in patients with POTS and, thus, potentially affecting the perceived discomfort from exercise, leading to possible avoidance, studies failed to demonstrate a higher prevalence of generalized anxiety and panic disorders in patients compared to the controls [8,9].

Other etiologies of POTS have been implicated more recently, including autoimmunity and possible neuroinflammation [10,11]. Patients with POTS were found to have a higher prevalence of various non-specific autoimmune markers, including antinuclear antibodies, and comorbid autoimmune disorders than the general population [12]. More specific to the autonomic system, ganglionic N-type and P/Q type acetylcholine receptor antibodies, alpha 1, beta 1, and beta 2 adrenergic antibodies, muscarinic M2 and M4 antibodies, angiotensin II type 1 receptor antibodies, and opioid-like 1 receptor antibodies were discovered in patients with POTS [10]. Lastly, current studies underscore shared pathophysiologic mechanisms in POTS and Long COVID, including autoimmune, immunologic, metabolic, endothelial, mitochondrial, and gut microbiome alterations [13,14]. Cardiovascular and metabolic pathophysiologies leading to secondary cardiopulmonary deconditioning in people with post-acute sequelae of SARS-CoV-2 infection could also play a role in the development of post-COVID POTS and other autonomic manifestations in some patients [15].

The treatment of POTS typically consists of a multidisciplinary approach encompassing pharmacologic and non-pharmacologic therapies [16]; however, research and clinical trials on non-pharmacological management have been limited. Known as the Dallas or the Levine Exercise Program, this exercise training program was the first exercise regimen found to be beneficial in a subset of patients with POTS; however, nearly 60% of patients enrolled in that study were unable to complete the high-intensity exercise program [7,17]. Additionally, common comorbidities, such as myalgic encephalomyelitis/chronic fatigue syndrome (ME/CFS), hypermobile Ehlers–Danlos syndrome (h-EDS), hypermobility spectrum disorders (HSD), and mast cell activation syndrome (MCAS), were not considered in the Dallas protocol, which made the outcomes of the Dallas exercise training program only relevant to a small subset of patients, given that the vast majority of patients with POTS has at least one comorbidity. Similarly, in a study by Pederson et al., almost half of the participants with POTS that began an intensive hospital-based rehabilitation program did not complete it, and more than half of those who completed it found it to be of no benefit. Those with POTS and comorbidities fared worse than patients without comorbidities [18]. In a scoping review, Peebles et al. emphasized a paucity of studies on exercise in people with POTS, joint hypermobility, and related conditions and the need to develop large clinical trials exploring exercise for the management of patients with POTS and comorbidities [19].

Exercise and other non-pharmacological treatments are uniformly implemented in the management of POTS because they have minimal side effects, are cost-effective, and are readily available for patients. However, what type of exercise can be performed safely, as well as its duration, intensity, and frequency, remains largely unknown. Furthermore, patients with severe POTS who are home- or bedbound are unlikely to access outpatient physical therapy and rehabilitation programs due to significant difficulties with mobility and fatigue. Additionally, many patients would require caregivers to drive them to and from outpatient facilities, which presents additional financial and social burdens for patients and their families. Finally, the impact of the SARS-CoV-2 pandemic on this vulnerable patient population with chronic illness has resulted in many patients avoiding unnecessary trips to medical facilities where the risk of re-infection, and, therefore, Long COVID and post-COVID exacerbation, is elevated. With these issues in mind, in this narrative review, we discuss the available literature on exercise in POTS and present our own Dysautonomia Clinic home-based exercise approach for patients with POTS, which can be delivered remotely. Our clinical experience utilizing this program suggests that this individualized exercise approach is easily accessible, convenient, safe, and sustainable in most patients with POTS, including those with severe POTS who are home- or bedbound.

## 2. POTS and Exercise: What Is Known

In addition to the Dallas exercise program, the Children’s Hospital of Philadelphia POTS Exercise Program was developed by modifying the Dallas protocol to make it easier to follow [20]. Both programs follow a traditional graded cardiovascular exercise approach with the assumption that cardiovascular deconditioning is involved in POTS [21]. While these programs are uniformly recommended to patients, it is unknown which subset of patients truly benefits from these programs and what the magnitude of benefit is compared to the natural history or standard therapies that do not include a structured exercise program.

Although cardiovascular deconditioning has been considered to be the cause or a major mechanism of POTS, emerging evidence suggests that this is not the case and that POTS involves various mechanisms, including cerebral hypoperfusion and possible neuroinflammation [7,11]. Van Campen et al. found a decline in cerebral blood flow during orthostatic stress in 171 study participants regardless of their deconditioning level or tilt table test results [22]. They concluded that neither POTS nor ME/CFS are caused by deconditioning and that exercise therapy alone is unlikely to be effective in improving orthostatic intolerance symptoms. In another study, exercise intolerance in patients with POTS was predicted by an exaggerated submaximal exercise heart rate, which was exacerbated in those achieving less than 85% of predicted metabolic equivalents [23]. Keller et al. found that, during two-day sequential cardiopulmonary exercise testing, a group of ME/CFS individuals failed to produce measures during the second day that were comparable to the first day of testing in peak exertion at work, exercise time, VO2, and VCO2, along with other tested measures [24]. Importantly, they concluded that contributions to exertion intolerance in ME/CFS by disrupted cardiac, pulmonary, and metabolic factors implicates autonomic nervous system dysregulation of blood flow and oxygen delivery for energy metabolism [24]. Conversely, in a study of an unsupervised at-home training regimen, cardiovascular exercise was found to improve cardiovascular function and the quality of life in those individuals with POTS who were able to complete this program [25]. As in other studies on exercise in POTS, only about a half of the patients enrolled in the exercise program were able to complete it [25].

Another possible mechanism of exercise intolerance in patients with POTS and autonomic dysfunction may involve muscle abnormalities. Orthostatic changes in muscle excitability were found in patients with POTS but not in healthy controls, which may be associated with inadequate perfusion of the lower extremities [26]. Muscle abnormalities were also found in people with Long COVID when exercising, which included greater concentrations of amyloid-containing deposits during post-exertional malaise (PEM)—a worsening of symptoms, such as severe fatigue, flu-like symptoms, brain fog, and others, which can occur hours or days after minimal physical or mental activity [27]. Increased tissue damage was observed with small atrophic fibers and focal necrosis that increased after exercise. Study participants displayed a reduction in skeletal muscle mitochondrial enzyme activity, a lower exercise capacity, an increased accumulation of amyloid-containing deposits in skeletal muscle, signs of severe tissue damage, and a slowed exercise-induced T-cell response in skeletal muscles [27]. These physiological changes could also be present in POTS patients and need to be examined in future studies.

Another mechanism that may be involved in exercise intolerance is hyperventilation—an important mediator in the pathophysiology of POTS. Importantly, hyperventilation in POTS appears to be unrelated to anxiety or panic disorder [28]. In one study, 65% of individuals with POTS in a cohort of 77 patients were diagnosed with dysfunctional breathing [29]. Breathing techniques and biofeedback are major tools to promote parasympathetic system activation. Balancing the output of the two systems can allow an individual to manage the symptoms resulting from autonomic nervous system imbalance.

POTS can be comorbid with ME/CFS, especially in severe cases. Pacing, a highly beneficial technique to conserve energy and mitigate PEM, involves activity alternating with rest and can be beneficial in patients with POTS, especially in those with severe symptoms and/or comorbid ME/CFS. Producing movement requires energy expenditure, and, therefore, joint co-contractions and stabilization are essential to completing any movement and exercise protocols. It is important to implement pacing and joint stabilization and avoid energy deficit when beginning and maintaining an exercise routine [18]. While aiming for improvement in functional capacity, focusing on muscle strengthening and the recruitment of type I slow-twitch muscle fibers is a priority as individuals with POTS and other comorbidities tend to recruit more type II muscle fibers, which contribute to PEM [29,30]. Importantly, a failure to address PEM can lead to failure of the entire exercise protocol and the inability to continue with a physical therapy program, which can worsen a patient’s physical and psychological state instead of improving it [30].

There is a significant paucity of data concerning exercise regimens in children and teens with POTS compared to adults. In one study of 18 adolescent patients, it was found that, during exercise, left ventricular end-diastolic and stroke volumes were lower in POTS patients, and the peak heart rate was higher in POTS patients compared to the controls, while the exercise time was higher in the control group [31]. Despite a lack of randomized controlled trials of exercise in pediatric POTS, more than 50% of 227 responders in a pediatric POTS survey felt that aerobic exercise was effective in reducing their symptom severity, and about 20% thought that it was the most beneficial intervention [32].

Finally, in one recent randomized controlled trial of a semi-supervised exercise program of 26 patients with POTS, including those with h-EDS, vs. 23 patients with POTS assigned to the standard of care, exercise training resulted in greater improvements in aerobic fitness, orthostatic symptoms, and exercise tolerance when intensity and progression were personalized and delivered with minimal supervision compared to the standard-of-care group [33].

In summary, the currently available studies on exercise in POTS suggest the following:

(1) Deconditioning is not the cause of POTS, MECFS, or orthostatic intolerance;
(2) Exercise intolerance is associated with several pathophysiologic mechanisms, including cerebral hypoperfusion, decreased venous return, mitochondrial dysfunction with systemic oxygen extraction, muscle abnormalities, and others;
(3) Exercise training programs may be of benefit to some patients with POTS while other patients, especially those with severe POTS and comorbidities, will need different, low-impact exercise approaches;
(4) These approaches should encourage manageable physical activity and movement via individualized, non-structured, and carefully designed exercise regimens that can lead to positive outcomes while preventing harm.

## 3. Individualized Approach to Exercise

We utilized the available literature on exercise, physical therapy, and rehabilitation programs in patients with POTS and our own clinical experience, which is skewed toward patients with moderate-to-severe POTS and comorbidities, given our referral base. By providing specialty care to patients with POTS and other common autonomic disorders, we gained further understanding and clinical experience that resulted in the development of our own unique approach to exercise. We found this approach to be beneficial in many patients with moderate-to-severe POTS via the optimization of orthostatic tolerance, energy level, and exertional capacity to yield improvements in movement and activities of daily living (ADLs).

Physical activity of moderate intensity, 150–300 min per week via brisk walking or cycling, and muscle-strengthening activity at least twice a week are recommended for all adults by the United States Health and Human Services (US HHS), including for adults with chronic health conditions and disabilities, with the specification that these recommendations apply to those individuals with chronic conditions who are able to exercise [34]. The guidelines add that, if adults with chronic conditions or disabilities are not able to meet the recommended guidelines, they should engage in regular physical activity according to their abilities and should avoid inactivity [34]. The last recommendation is particularly important for patients with POTS and those with exercise intolerance in general, many of whom may not be able to accomplish daily 25–30 min exercise of a moderate intensity. We therefore incorporated the US HHS recommendations for individually tailored physical activity and avoidance of inactivity to our patients’ abilities in our Dysautonomia Clinic individualized exercise approach.

As stated above, existing exercise protocols have a low success rate for individuals with POTS, especially when accompanied by severe symptoms and comorbidities. Therefore, the focus of our program is on treating the entire body and functional impairments in an individualized and targeted exercise approach—not the one-size-fits-all model commonly used in existing structured exercise programs. Our goal is to reduce inactivity and immobility and encourage physical activity and movement in all patients with POTS and comorbidities, including home- and bedbound patients.

Extensive patient education is provided on how the ANS works in controlling the body’s automatic functions in order to empower the patient to engage in their own exercise program and ensure compliance. How the sympathetic and parasympathetic systems work together to maintain homeostasis and a thorough description of the pathophysiology of the ANS and the physical characteristics associated with the activation of this system are integral to understanding how sympathetic overactivity is manifested physically. Education and knowledge of why symptoms occur are key components in learning to manage them. Facilitating parasympathetic nervous system activation and reducing sympathetic overactivity are among the mechanisms that can improve autonomic symptoms. An emphasis on these ideas enables patients to gradually gain control of physical function in order to tolerate ADLs. Diaphragmatic breathing techniques and other methods to increase parasympathetic activation are also utilized for symptoms of POTS [29].

Prior to any exercise initiation, we conduct a detailed pre-exercise evaluation of the patients, which includes a complete medical history, a physical exam, and a 10 min stand test with measurements of blood pressure and heart rate in the supine and standing positions (Table 1). A Beighton score for joint hypermobility, a 6 min walk test, and the Borg Scale can also be utilized (Table 2) [35].

**Figure 1:**
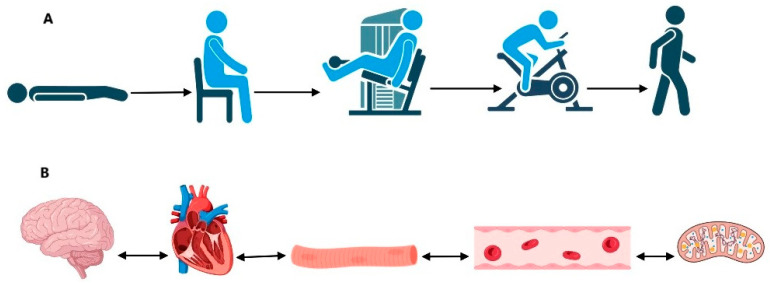
The goals of exercise progression from supine to standing (**A**) and factors that may affect the ability to exercise (**B**) in patients with POTS. (**B**) Pictured are the brain, the heart, the skeletal muscle, blood flow through a capillary, and a mitochondrion, all of which may play a role in POTS-associated exercise intolerance, one may affect the function of the other.

Remote delivery of the program allows individuals to access appropriate treatment from where they are located, practice energy conservation by not having to travel to appointments, and benefit from one-on-one conversation without the distractions typically present in an outpatient clinic. This method allows for specific individualization and the ability to modify interventions and education based on the patient’s presentation that day. Our trained physical therapist completes 30–45 min sessions remotely via video platforms, with a typical frequency of once a week. For example, the goal of the sessions for patients with severe POTS is to gradually progress first to sitting comfortably and then accomplishing a standing activity in order to return to ADLs without becoming severely symptomatic during or after the activity. For those patients who are bedbound, we recommend first increasing the incline of the bed, then reclining instead of lying flat, then sitting with legs up, then sitting with legs down, and then comfortably walking around the room (Figure 1). Increasing the duration of these positions and activities gradually is the key to reducing severe orthostatic symptoms and PEM. A graded introduction of a combination of aerobic activity and light strengthening is the basis of our exercise approach [20]. The Borg Scale is utilized as there is no uniform guideline to otherwise determine the amount of exercise a patient can tolerate and should be prescribed [35]. The Borg Scale of perceived exhaustion allows us to quantify a patient’s exertion to ensure that they are exercising at an appropriate level. The patient’s ability to complete an exercise routine at home without causing symptomatic worsening is essential to improving overall function and eventually progressing to a more traditional exercise routine [20].

A general overview of our exercise approach is conceptualized in Figure 1 and Table 3. First, a baseline assessment of core activation and proximal stability is completed via active movements that can be observed through video platforms by our physical therapist. When other comorbidities, such as h-EDS or HSD exist, the plan is modified to focus more on proximal stability and joint co-contractions to facilitate joint control with movement. Appropriate joint stability in proximal joints, such as the shoulders and hips, is key to having functional movement patterns in order to complete ADLs and community ambulation or activities. Furthermore, the proper pre-treatment of individuals that have MCAS is important to decrease the potential for adverse reactions due to heat and other exercise-induced triggers for mast cell activation syndrome. Pre-treatment with antihistamine medications prior to exercise may be necessary and should be discussed with the treating physician with expertise in POTS and comorbidities. Understanding the pathophysiology of POTS, h-EDS/HSD, and MCAS and how exercise might temporarily increase symptoms with this commonly observed triad in clinical practice is important when recommending physical activity to patients with these triads.

The patient’s understanding of the quality of movement versus the quantity improves compliance with functional activities or exercises. The Borg Scale is used to pace activities in order to take into account post-exertional malaise (Table 2). It is also commonly used in physical therapy practice to determine the rate of perceived exhaustion (RPE) and guide exercise in patients appropriately [4,18]. The BORG Scale is helpful in giving an objective component to how an individual feels while completing tasks such as walking to the bathroom, standing to cook a meal, or other necessary functional activities [35].

Since a significant subset of patients with severe POTS may have comorbid ME/CFS, education on PEM and the difference between this and delayed onset muscle soreness is crucial to their successful return to activities. Patient understanding of pacing is also an integral part to continuing forward momentum to improve function. Pacing and individualization are critical in considering PEM in order to continue to improve function and tolerance for activities. Once pacing is mastered, increased strengthening and aerobic exercise can be added. An emphasis on joint co-contraction and stabilization is prioritized due to many individuals with POTS having joint hypermobility. Focusing on core stabilization and strengthening proximal muscle groups in the beginning set the individual up for more challenging exercises later, especially those with dynamic and long lever components. This also addresses PEM, which is common in this patient population, as the muscles utilized for core stabilization, such as the transversus abdominis, have an increased concentration of type I muscle fibers, which reach a state of fatigue slower than other types.

Table 3 summarizes the beginning, intermediate, and advanced components of our POTS exercise program. Our focus on core stabilization is integral for the long-term goal of improved mobility. Previous protocols that focused on aerobic conditioning did not take into account the strength and stability needed to perform higher-level activities. There needs to be a focus on proximal stability and core activation prior to performing dynamic activities; this is especially important given a high prevalence of comorbid h-EDS and HSD in patients with POTS. An improved core stability also assists the person in energy conservation as other muscle groups do not have to facilitate stability when their mode of action is to produce movement. Joint co-contractions and stability are key to improving tolerance of the dynamic movements that are required for the completion of daily activities.

ADLs, such as toileting, dressing, or bathing, as well as instrumental activities of daily living, such as cooking, cleaning, or driving, are often difficult aspects of daily life for patients with severe POTS. A significant subset of these patients require assistance with self-care, and many patients need help completing basic household tasks. We encourage our patients to incorporate frequent short bouts of activity, like walking to and from rooms of a house or apartment, as a means to improve endurance. Understanding and using the Borg Scale, as described above, is a mode to assess the degree of exertion during any activity. Routine activities, such as ascending/descending stairs, walking laps in a living space, and progressive standing at a counter, are interventions that are available and accessible for people to complete as tolerated. As stated above, the goal for all patients is to be able to safely accomplish these routine ADLs. Importantly, we believe that patients need to be able to tolerate these activities prior to beginning a structured and more intense conditioning program. In our experience, many patients are unable to engage in aerobic exercise training programs if they are unable to tolerate routine ADLs. Achieving tolerance and mobility to accomplish the simple tasks of being able to safely navigate one’s living environment is necessary and critical before considering any structured exercise program. In fact, for some patients, achieving tolerance and mobility to safely tolerate ADLs without PEM would be the ultimate goal, while an additional structured exercise program may be unattainable.

Once an individual is able to tolerate ADLs, dosed exercise can be integrated to improve overall lifestyle and function. Emphasis is placed on taking into account what activities have already been completed in the day prior to embarking on the structured aerobic activity. Exercise on a recumbent bike, a rowing machine, walking in the pool, swimming, or elliptical exercise are good options for aerobic activity in patients with POTS and comorbidities. Exercise in the water may be especially helpful for patients with POTS and comorbid h-EDS and HSD, given the benefits of water on both orthostatic intolerance and joint pain. The time on the equipment or in the pool is gradually increased while starting at a reasonable time that is individualized. The duration of the aerobic exercise is slowly increased, and then the resistance or workload is increased. Individuals are encouraged to use a journal, symptom log, or other modes of documentation in order to properly dose the exercise. At times of symptom flares, the duration and resistance or aerobic activity altogether might need to be modified or avoided, and the beginning supine core stabilization exercise and parasympathetic activation activities should be implemented in order to manage symptoms.

We emphasize to all patients that this is not a linear process and that peaks and valleys are expected in order to reach the target goal of an improved functional status (Table 3). Helpful facts on exercise are discussed and reinforced over the course of the program to ensure patient understanding, empowerment, and compliance (Table 4). Many patients have been told by their physicians that their exercise intolerance is caused by poor effort, fear, or a lack of motivation: these assertions are not supported by robust clinical evidence and can breed resentment, negativity, self-blame, and poor compliance on the part of the patient. Therefore, it is important to emphasize that the patient did not cause their own exercise intolerance because it is a pathophysiologic problem intrinsic to their autonomic dysfunction, but that maintaining some type of exercise and movement is still important to avoid further deterioration and disability (Table 4).

Finally, it is important to add that some patients, usually those with extremely severe, intractable symptoms, will not be able to maintain any exercise routine despite their best efforts and those of their healthcare professionals. In this case, we continue to recommend engaging in some movement, which may include walking around the room, leg lifts which can be carried out in bed, stretching, and breathwork. A trusting and supportive healthcare practitioner–patient relationship is at the core of optimal clinical care, especially for severely disabled patients with complex chronic illnesses. Our experience working with bedbound people with POTS has shown us that blaming the patient, attaching psychological or psychiatric labels, pushing them beyond their physical limits, or dismissing the patient from practice because they were unsuccessful in increasing physical activity—actions which, unfortunately, are not uncommon in clinical practice—are detrimental and damaging to the patient and their trust in the healthcare system. For these and other reasons, many severely disabled patients with POTS and ME/CFS end up being medically neglected, which leads to their further physical and psychosocial deterioration. Thus, we strongly encourage clinicians, physical therapists, occupational therapists, psychologists, and social workers to continue their therapeutic relationship with these patients in a supportive and non-judgmental manner.

With our individualized home-based exercise approach delivered remotely, we observed that patients with POTS of various severity demonstrated an increased tolerance for family life activities, a return to community access in short periods of time, and an increased ability to engage in work, school, and social events. Since the program is administered by a licensed physical therapist remotely, patients with POTS are able to engage safely in movement and exercise under the guidance of a trained professional while bypassing the energy requirements and caregiver time needed to reach a physical therapist outside of the home, thus allowing for better pacing and investment of their energy into the actual exercise program instead of travel and wait times at an outpatient clinic. Additionally, given a persistent risk of COVID-19 and other respiratory infections in the community and at medical facilities, especially with indoor masking no longer being a requirement, our home-based exercise approach presents a safe and convenient option. Its remote delivery reduces the transmission of respiratory infections and the risk of Long COVID and other post-acute infection complications in this vulnerable patient population.

## 4. Conclusions

Given that the dropout rate in existing exercise training programs that are widely recommended by healthcare professionals is high and that exercise intolerance is a key feature in patients with POTS, an individualized home-based exercise approach may present a viable, convenient, easily accessible, safe, and sustainable exercise option that most patients, including those with severe POTS who are home- or bedbound, are able to engage in. At Dysautonomia Clinic, we focus on an individualizing exercise approach based on the patient’s current symptoms and comorbidities to maximize patient participation, improvement in symptoms and overall quality of life, and ensure sustainability and safety.

## 5. Future Direction

**Figure 2:**
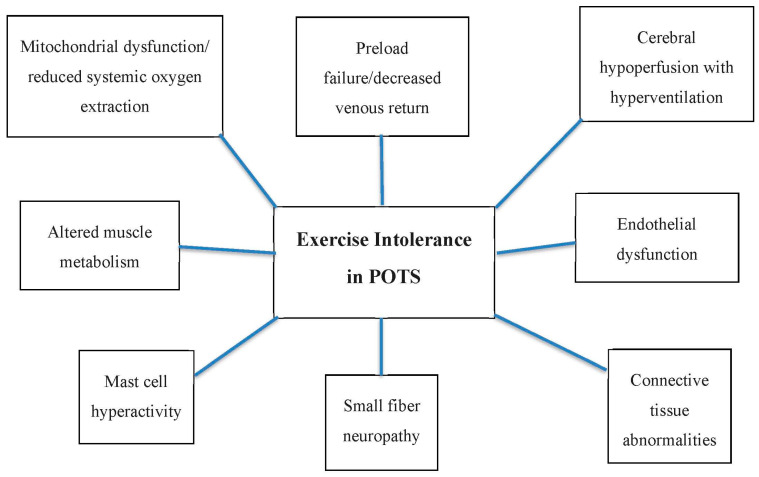
Pathophysiology of exercise intolerance in POTS: factors which may cause or contribute to reduced ability to exercise.

Many questions regarding exercise in patients with POTS and comorbidities remain unanswered. First, there are no guidelines or specifications on what target heart rate is safe or appropriate to recommend. Anecdotally, we and others advise our patients to aim for a target heart rate of 70–75% of a maximum heart rate, but establishing an alternative formula for this specific patient population, based on robust data, is clearly needed because the standard formula of 220 bpm minus one’s age is likely neither realistic nor appropriate for patients with tachycardia and exercise intolerance. Second, the pathophysiology of exercise intolerance and the reasons for reduced systemic oxygen extraction with decreased VO2 max remain elusive: the interplay between mitochondrial dysfunction, endothelial abnormalities, small-fiber neuropathy, mast cell mediators, and other factors need to be studied in detail (Figure 2). Third, the nature of PEM and how to mitigate its effect so that patients may routinely engage in exercise need to be determined. Fourth, personalized and safe multidisciplinary physical and occupational therapy approaches for patients with severe orthostatic and exercise intolerance need to be developed and validated in large studies. Finally, a randomized controlled trial of Dysautonomia Clinic exercise approach vs. standard therapy or natural history of POTS is needed to quantify the impact of our program on objective and subjective outcome measures, including quality of life and autonomic symptom burden, especially when millions of people are suffering from post-COVID POTS in the context of Long COVID.

## Figures and Tables

**Table 1:** Pre-exercise evaluation of patients with POTS.

Complete history and physical exam performed by a healthcare practitioner Self-report of asymptomatic sitting and standing time The Borg Scale Standing and supine blood pressure and heart rate, including a 10 min stand test for assessment Oxygen saturation via pulse oximetry 6 min walk (or a plausible substitute) Beighton score for joint hypermobility

**Table 2:** The Borg Exertion Scale used to quantify patients’ rate of perceived exertion (RPE) [35].

10	Max Effort	Out of breath, unable to talk, impossible to continue
9	Very Hard	Very difficult to breathe, can only speak a few words
7–8	Hard	Uncomfortable, can speak a sentence or two
4–6	Moderate	Breathing heavy but can hold a conversation, becoming more challenging
2–3	Light	Could continue for hours, easy to breathe and hold a conversation
1	Very Light	Minimal exertion

**Table 3:** Key components utilized in the Dysautonomia Clinic exercise approach to POTS.

Initial Component	Intermediate Component	Advanced Component
Core stability	Dynamic stabilization	Functional activities
Parasympathetic activity enhancement	Using breathing to improve tolerance for function	Functional activities with minimal symptoms
Pacing of activity	Increase time of activity	Progression to aerobic activity while still pacing

**Table 4:** Helpful facts on exercise for patients with POTS.

POTS is not caused by deconditioning but can lead to deconditioning. Exercise intolerance is a physiologic problem. You did not cause it. Exercise is important to maintaining function. It is healthy and natural to move your body. Immobility and continuous bed rest will make POTS worse in the long run. If your heart rate is high during or after exercising, you will get thought it, and your heart rate will decline with time. Even if you overdo it with exercise, you will eventually recover. You cannot permanently damage your body by doing or overdoing your exercise routine even if your POTS symptoms become temporarily worse. If you have both POTS and ME/CFS, we will adjust your exercise recommendations to include pacing and minimizing post-exertional malaise. Even if it is not improving your POTS symptoms, you still benefit from exercise in other ways. Exercise conducted at your own pace improves your muscles, bones, brain, heart, lungs, blood vessels, digestive system, metabolism, sleep, and mental health. Even a few minutes of exercise per day are better than none. Hydrate before and after exercise. Keep room temperature cool during and after exercise to minimize overheating. Talk to your doctor about taking extra medication before or after exercise if needed. Listen to your body but continue pushing yourself by starting low and going slow.
